# Effects of different forage proportions in fermented total mixed ration on muscle fatty acid profile and rumen microbiota in lambs

**DOI:** 10.3389/fmicb.2023.1197059

**Published:** 2023-07-13

**Authors:** Mingjian Liu, Zhijun Wang, Lin Sun, Yu Wang, Junfeng Li, Gentu Ge, Yushan Jia, Shuai Du

**Affiliations:** ^1^Key Laboratory of Forage Cultivation, Processing and High Efficient Utilization of Ministry of Agriculture and Rural Affairs, Inner Mongolia Agricultural University, Hohhot, China; ^2^Key Laboratory of Grassland Resources of Ministry of Education, Inner Mongolia Agricultural University, Hohhot, China; ^3^Inner Mongolia Academy of Agricultural and Animal Husbandry Sciences, Hohhot, China; ^4^Inner Mongolia Yili Industrial Group Co., Ltd., Hohhot, China

**Keywords:** oat, alfalfa, fermented total mixed ration, meat quality, rumen microbiota

## Abstract

**Objective:**

The objectives of this study were to evaluate the effects of different forage proportions in the fermented total mixed ration (FTMR) on growth performance, muscle fatty acid profile, and rumen microbiota of lambs.

**Methods:**

Thirty 6-month-old small tail Han sheep × Ujumqin lambs with initial body weight (BW) of 27.8 ± 0.90 kg were selected for the test and divided into two groups of 15 sheep in each treatment (three pens per treatment and five lambs per pen) according to the principle of homogeneity. Two isoenergetic and isonitrogenous diets were formulated according to the NRC. The diet treatments were designed as (1) OH treatment containing 25% alfalfa hay and 35% oat hay, and (2) AH treatment containing 35% alfalfa hay with 25% oat hay. The forage-to-concentrate ratio for both diets was 65: 35 (DM basis). Three replicates were randomly selected from each treatment to determine growth performance, fatty acid profile and rumen bacterial communities in lambs.

**Results:**

Results revealed no statistically significant (*p* > 0.05) differences in dry matter intake and average daily gain between the two diet groups. Cholesterol and intramuscular fat were significantly (*p* > 0.05) higher in the AH group, while no statistically significant difference (*p* > 0.05) was found in pH24 value. The muscle fatty acid compositions of lambs were obviously (*p* < 0.05) influenced by the diet treatments. Compared with the OH group, the C16:1, C17:0, and C20:3n6 contents were higher (*p* < 0.05) in the AH group, whereas the content of C18:1n9c, C20:1, C18:3n3, and C22:6n3 was obviously (*p* < 0.05) increased in the OH group. The monounsaturated fatty acid (MUFA) contents were significantly higher in the OH group, whereas no significant differences (*p* > 0.05) were detected in saturated fatty acid (SFA) and polyunsaturated fatty acid (PUFA) contents among the two diet treatments. Bacterial composition was generally separated into two clusters based on principal coordinate analysis, and the OH group had a higher Shannon index. The relative abundance at the genes level of the *Rikenellaceae_RC9_gut_group* was obviously (*p* < 0.05) increased in the AH group and the relative abundances of *Prevotella_1, Fibrobacter*, and *Bacteroidales_UCG_001_unclassified* were obviously (*p* < 0.05) enriched in the OH group. Integrated correlation analysis also underscored a possible link between the muscle fatty acid compositions and significantly altered rumen microbiota.

**Conclusion:**

Overall, oat-based roughage in FTMR could promote a beneficial lipid pattern in the Longissimus lumborum muscles of lambs. These findings provide a potential insight into diet effects on fatty acid profile and the rumen microbiome of lambs, which may help make decisions regarding feeding.

## Introduction

Alfalfa (*Medicago sativa* L.) is a promising forage used for animals worldwide due to its strong adaptability and good nutritional value (Zhao et al., 2021). However, the higher cost of alfalfa might affect economic efficiency (Wang et al., 2021; Chávez et al., 2022). Thus, a high-priority task is to find suitable forages to replace alfalfa hay in order to meet the needs of the herbivorous animal industry.

Oat (*Avena sativa* L.) has high protein and digestible fractions, can grow well in the cooler temperatures of spring and fall, and is an important part of feedstuff for ruminants (Harper et al., 2017; Jia et al., 2021). Previous research has demonstrated that oat hay has been widely used due to its advantages of higher apparent digestibility of fiber, which can balance the stable state of the rumen internal environment in animals (An et al., 2020). Zou et al. (2018) demonstrated that partially replacing alfalfa hay with oat hay in total mixed rations (TMR) could promote nitrogen utilization. However, TMR is associated with a high level of aerobic deterioration and needs to be freshly prepared prior to each use, which limits its use in some farms due to labor shortages (Du et al., 2022). Previous research has demonstrated that fermented total mixed ration (FTMR) could be used as a diet in animal production (Kumagai et al., 1983). In addition, FTMR could improve total tract apparent digestibility while decreasing fecal N excretion, which enhances economic benefits and reduces environmental pollution (Zhang et al., 2020). Furthermore, Zhao et al. (2020) confirmed that bamboo (*Bambusoideae*) as a roughage source of FTMR had no adverse effect on fermentation quality while improving aerobic stability.

The rumen serves as a natural bioreactor and is occupied by a highly diverse and dense microbiota population consisting of bacteria, fungi, archaea, and protozoa (Carballo et al., 2021). The rumen is responsible for the ruminant's ability to convert fibrous plant materials and indigestible plant mass into energy and protein (Carrasco et al., 2017). The rumen microbial population ferments feedstuffs and converts fibrous-rich plant materials into microbial proteins. This unique microbial ecosystem provides ~70% of the daily energy requirement for ruminant needs and improves the growth and performance of the host (Krause et al., 2020; Du et al., 2022). Additionally, rumen bacteria are involved in biological hydrogenation and isomerization *in vivo*, which may eventually affect muscle fatty acid (FA) deposition by changing the FA composition of the rumen digesta (Enjalbert et al., 2017). The fatty acid composition of meat is closely related to human health benefits, and the excessive consumption of saturated fatty acid (SFA) will increase the risk of cardiovascular disease (Abuelfatah et al., 2016). A previous study suggested that replacing SFA with polyunsaturated fatty acid (PUFA) may be beneficial to human health, which can be achieved by altering rumen microbial metabolism (Zhu et al., 2022). In turn, the host provides an anaerobic and substrate-rich environment for microbes to thrive (Ellison et al., 2017). Generally, bacteria are the most important players among rumen microorganisms during feed biopolymer degradation and fermentation (Welkie et al., 2010). However, the composition and distribution of rumen microflora are affected by many factors, especially diet, season, environmental temperature, and the development and growth stage of the host animals (Cui et al., 2022). Previous research showed that dietary composition was the main factor affecting rumen microbial compositions and functions (Liu et al., 2022). Li et al. (2020) provided evidence that the rumen microbial community and their functions could be dramatically affected by different diets and further affect the metabolism of fatty acids by ruminal microorganism biohydrogenation in the muscle of animals. Studies have shown that the 20% changes in feed efficiency could be explained by the rumen microbiome (Liu et al., 2022). Therefore, a greater understanding of the functions of rumen microbiota is necessary to investigate the mechanisms involved in the effects of dietary composition on the formation and improvement of the quality of meat produced by animals. However, data on the influences of different forage proportions in FTMR on the fatty acid profile and ruminal microbiota are still lacking.

We hypothesized that increasing the proportion of oat hay in FTMR would provide sufficient physical active fiber to enhance rumen functions and the meat quality of lambs. Therefore, this study aimed to evaluate the effects of forage proportion in FTMR on muscle fatty acid composition and the rumen microbiota of lambs.

## Materials and methods

### Animals, diets, and experimental design

The study protocol selected for this experiment was based on the Institutional Guidelines for Animal Experiments and the Regulations for the Administration of Affairs Concerning Experimental Animals of the College of Grassland, Resources, and Environment, Inner Mongolia Agricultural University, Hohhot, China. All the experimental protocols carried out in this study were approved by the Animal Care Committee of Inner Mongolia Agricultural University. The feeding experiment was carried out at Inner Mongolia Hongpeng Technology Co. Ltd (Balin Left Banner, Chifeng, China). A total of 30 small-tail Han sheep × Ujumqin sheep crossed uncastrated male lambs weighing ~27.8 ± 0.90 kg were randomly allocated to the two diet groups, with 15 sheep in each treatment (three pens per treatment and five lambs per pen) according to the principle of homogeneity. The pens were separated into five single stalls (0.8 × 1.3 m). Two isoenergetic and isonitrogenous diets were formulated according to the NRC (NRC, 2007; Nasehi et al., 2018). The forage-to-concentrate ratio for both diets was 65:35 (DM basis) to meet the essential energetic and nutritional requirements of the experimental lambs. The gross energy (GE), energy in feces (FE), energy in urine (UE), and energy in gaseous products of digestion (Eg) were analyzed using an oxygen and nitrogen analyzer (IKA Works GmbH and Co., Staufen, Germany). Metabolizable energy (ME) was estimated following the procedure reported by Freer et al. (2007), and the formula was as follows: ME = GE – FE – UE – Eg. The composition and nutrition of the diets are listed in Supplementary Table 1. The FTMR was prepared with alfalfa hay, oat hay, corn stover, natural forage, and concentrate mixtures provided by Chaoyue Feed Co. Ltd. The experimental treatments were designed as (1) OH containing 25% alfalfa hay with 35% oat hay and (2) AH containing 35% alfalfa hay with 25% oat hay. The diets were prepared as follows. The forage samples were chopped into ~1–2 cm and mixed according to the above ratios, and then adjusted for moisture to 500 g/kg using a water sprayer after additives had been dissolved in water. The additives were compound bacterial agents (containing *bacillus subtilis* R2 and *bacillus subtilis* N10) purchased from Hebei Zhong bang Biotechnology Co. Ltd. (Strong brand, Hebei, China) and were added in proportion to 1 g/kg of fresh TMR. FTMR was placed in fermentation bags (55 × 85 cm) equipped with a one-way exhaust valve, compacted, and sealed at the mouth of the bag, and all samples were stored indoors (~15°C) for 60 days. The experiment was performed over 75 days, with the first 15 days for adaptation. During the adaptation period, the animals also received their respective test diets. The lambs were fed twice daily (7: 00 h a.m. and 5: 00 h p.m.), and no more than 10% of refusals were allowed. The lambs were provided with free access to drinking water and fed *ad libitum* on their respective FTMR diets.

### Sample collection and processing

The weight of feed delivered and refused was registered every day to evaluate the dry matter intake (DMI). The weight of all lambs was assessed after their adaptation period and every 2 weeks thereafter. The initial body weight and final body weight were measured to estimate the average daily gain (ADG). Following the experimental tests, the experimental lambs were provided with free access to drinking water and fasted for 12 h, then stunned electrically, and exsanguinated to death by severing the jugular vein at a butchery. The slaughter procedures were performed according to operating procedures of livestock and poultry slaughtering for sheep and goat (NY/T 3469-2019, Ministry of Agriculture, China) (Ministry of Agriculture, 2019). The muscle samples of *Longissimus lumborum* were isolated from the right side of the carcass to determine pH value, cooking loss rate, and dripping loss rate, and the part of the isolated sample was immediately stored at −20°C in a freezer until further assay of chemical composition, meat quality, and fatty acid profile. In addition, rumen fluid samples of all lambs were collected immediately after slaughtering, and six ruminal fluid samples were randomly selected from each group for further measurement. Approximately 50 ml of rumen fluid was gathered from each lamb by straining the rumen content with four layers of cheesecloth. The rumen fluid samples were immediately frozen in liquid nitrogen and then stored in a cryogenic refrigerator at −80°C for 16S rRNA sequencing analysis.

### Feed composition analysis

Feed samples were dried for 48 h in a convection oven at 60 to 65°C until constant weight to calculate dry matter content (DM) and then ground with a sample mill (1 mm screen) for further analysis of crude protein (CP), organic matter (OM), neutral detergent fiber (NDF), acid detergent fiber (ADF), and ash. DM, CP, OM, and ash were analyzed following the AOAC (2005) standard procedures (925.04, 954.01, 920.39, and 942.05, respectively). ADF and NDF contents were measured based on the methods previously published by Van Soest et al. (1991). Ten grams of FTMR samples were randomly selected and further mixed with 90 mL sterilized saline and homogenized in a blender for 2 min to extract the fermentation broth. After that, the bacterial solution was diluted from 10^−1^ to 10^−5^ to count the number of microorganisms. The amount of LAB and aerobic bacteria was calculated with the MRS medium base (MRS) medium and nutrient agar, respectively. The pH value of the FTMR extract was measured immediately with a calibrated glass electrode pH meter (STARTED 100/B, OHAUS, Shanghai, China). The concentrations of organic acids were characterized based on high-performance liquid chromatography (HPLC) following the method reported by You et al. (2021). Ammonia nitrogen (NH_3_-N) was assayed by the phenol-hypochlorite procedure according to the methods of Broderick and Kang (1980).

### Meat quality analysis

Association of Official Analytical Chemists methods (AOAC, 2005) were used to assess the contents of moisture, protein, cholesterol, intramuscular fat, and ash. The pH_24_ value of the *longissimus lumborum* muscle samples was estimated after 24 h (stored at 4°C) by a pre-calibrated portable pH meter equipped with a glass electrode shaped to easily penetrate meat (STARTED 100/B; Ohaus, Shanghai, China). The cooking loss rate of the *longissimus lumborum* muscle samples was calculated by taking the difference between the initial uncooked weight and the final weight of the sample after cooking following the recommendations of the American Meat Science Association (Seman et al., 2017). The samples were stored in inflated plastic bags at 4°C for 24 h. The dripping loss rate was represented by the ratio of weight loss (Honikel, 1998). The fatty acids of *longissimus lumborum* samples were tested by gas chromatography (GC) of fatty acid methyl esters (FAMEs). The GC was equipped with a fused silica capillary column (crosslinking bonded stationary phase containing 50% propyl, 60 mm × 0.25 mm × 0.25 μm), split injector, and flame ionization detector fitted with a Galaxie Chromatography Data System (Version 1.0 software). The oven was programmed as follows: initial temperature of 130°C for 1 min, which was first increased to 170°C at 6.5°C/min, then increased to 215°C at 2.75°C/min and held at that temperature for 12 min, and finally, the temperature was further increased to 230°C at 4°C/min and held at that temperature for 3 min. The injector and detector temperatures were 270 and 280°C, respectively. Helium was used as a carrier gas at the column flow of 0.8 ml/min and a split ratio of 50:1. The methylation of fatty acids in meat samples was carried out based on the procedure of AOAC (2005). The compositions of muscle saturated fatty acid (SFA), monounsaturated fatty acid (MUFA), and polyunsaturated fatty acid (PUFA) were obtained from individual fatty acid percentages.

### Bacterial DNA extraction, polymerase chain reaction amplification, and 16s rRNA sequencing

The microbial DNA of six rumen fluid samples was isolated using E.Z.N.A. ^®^Stool DNA Kit (D4015, Omega, Inc., USA) and then eluted with elution buffer (50 μL) following the manufacturer's instructions. The V3–V4 region of the 16S rRNA gene was amplified from extracted DNA as previously described using the universal primers of 341 (5′- CCTACGGGNGGCWGCAG-3′) and 805 (5′-GACTACHVGGGTATCTAATCC-3′) (Logue et al., 2016). The PCR products were extracted and purified by 2% agarose gel electrophoresis and AMPure XT beads (Beckman Coulter Genomics, Danvers, MA, United States), respectively. The sequencing library was prepared using the gDNA samples applying the Illumina library quantification kit (Kapa Biosciences, Woburn, MA, United States). The amplicon libraries were evaluated and characterized for size distribution and number using an Agilent 2100 Bioanalyzer (Agilent, United States). The sample libraries were sequenced using the NovaSeq PE250 platform. Sequencing data for the 16S rRNA gene sequence of the rumen samples in the two FTMR diet groups were uploaded and stored in NCBI BioProject, and the accession number can be found under PRJNA888233.

### Bioinformatics analysis

The 16S rRNA gene sequences were performed using an Illumina NovaSeq platform according to the recommended vendor's instructions (LC-Bio Technology Co. Ltd., Hangzhou, Zhejiang Province, China). Paired-end reads were assigned to samples based on their unique barcode and truncated by cutting off the barcode and primer sequence. FLASH (v1.2.8, http://ccb.jhu.edu/software/FLASH/) was used to merge the paired-end reads. Quality filtering was performed on the raw reads under specific filtering conditions to obtain high-quality clean reads according to fqtrim (v0.94, http://ccb.jhu.edu/software/fqtrim/), and Vsearch (v2.3.4) was performed to filter the chimeric sequences. QIIME2 (v2019.7) was selected to import and process the sorted reads for bioinformatics analysis. The imported paired reads were quality filtered, denoised, and merged by plugin DADA2 (v3.11) to produce the amplicon sequence variants (ASV) feature table. The taxonomy classification was carried out using the q2-feature-classifier, a taxonomic classifier plugin for the QIIME 2 microbiome analysis platform (https://qiime2.org/) based on the scikit-learn naive Bayes classifier. After that, a SILVA database (Release 138, http://www.arb-silva.de) was used to assign taxonomy to filtered ASVs, predicting 99% identity of bacteria and representative sequences. To determine the species diversity in each sample of the two treatments, alpha diversity and beta diversity analyses were conducted using the procedure of plugin q2-diversity in QIIME2 (v2019.7). To assess bacterial communities between individuals and groups in the current research, weighted UniFrac outputs were evaluated and visualized using PCoA. The communities that showed statistical differences among the two groups were identified using PERMANOVA (vegan 2.5.4). Treatment-dependent features were identified using LEfSe. A size-effect threshold of 3.0 on the logarithmic LDA score was used to identify discriminating taxa, as proposed by Segata et al. (2011). Using the Mann–Whitney *U*-test, the differences in relative abundance were identified between groups. To illuminate the interactions between microbes and meat quality, the relationships between the top 20 genera bacterial community and meat quality were visualized by Pearson's correlation heatmap, which was performed using R Core Team (2014) after ranking and normalizing the correlation matrix. Phylogenetic Investigation of Communities by Reconstruction of Unobserved States 2 (PICRUSt2) was used to infer the rumen microbiota functional pathways based on the information from the KEGG database (Douglas et al., 2020). Bar graphs were drawn with the software GraphPad Prism 9 (San Diego, CA, United States).

### Statistical analysis

Analyses of feed nutrition, growth performance, meat quality, and muscle fatty acid composition were performed using SAS ver. 9.2 (SAS Inc, 2007 Cary, NC, USA). The statistical model of the SAS was as follows: Y = μ + α+ ε, where Y = observation, μ = overall mean, α = diet effect, and ε = error. Differences in means among the two groups were evaluated using an independent sample *t*-test, and a *p-value of* < 0.05 was considered to be statistically significant.

## Results

### Animal performance

**Figure 1:**
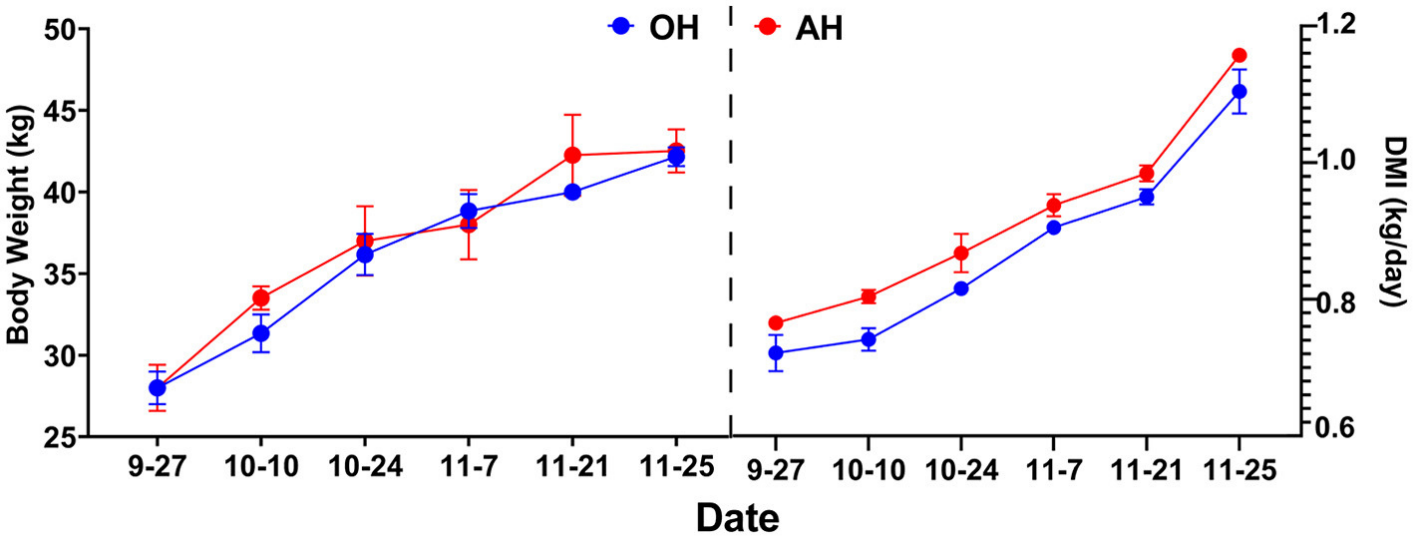
Dynamic changes of ADG and DMI every 2 weeks among different treatments. DMI, dry matter intake; OH, high oat percentages group; AH, high alfalfa percentage group.

The initial BW, final BW, ADG, and DMI of all lambs across the entire fattening trial are shown in Table 1. Interestingly, no statistically significant (*p* > 0.05) difference in final BW between these two different diet groups was observed. There were no significant (*p* > 0.05) differences observed in DMI or ADG between the two diet treatments. As shown in Figure 1, the dynamic changes in DMI and ADG followed the same trend and gradually increased in the two diet treatments.

**Table 1:** Growth performance of lambs feed fermented total mixed rations with different ratios of alfalfa and oat.

**Items**	**OH**	**AH**	**SEM**	***P*-value**
Initial BW (kg)	27.67	28.00	0.366	0.875
Final BW (kg)	41.00	42.51	0.569	0.267
ADG (g/day)	222.22	241.83	5.119	0.181
DMI (kg/day)	1.10	1.16	0.017	0.210

BW, body weight; ADG, average daily gain; DMI, dry matter intake; SEM, standard error of the mean. Means within the same rows with different letters are significantly different (*p* < 0.05). SEM, standard error of means. OH, high oat percentages group; AH, high alfalfa percentage group.

### Meat quality

Table 2 shows that there was no statistically significant (*p* > 0.05) difference in pH_24_ value between the two diet treatments. The dripping loss rate of the *longissimus lumborum* muscle was obviously (*p* < 0.05) increased in the AH group, whereas no significant (*p* > 0.05) qualitative difference was detected in the cooking loss rate between the two diet groups. No statistically significant (*p* > 0.05) differences were observed in muscle moisture or protein of lambs in the two diet treatments. Compared with the OH group, the cholesterol and intramuscular fat were significantly (*p* < 0.05) higher in the AH group, while ash was significantly (*p* > 0.05) lower.

**Table 2:** Meat quality of lambs feed fermented total mixed rations with different ratios of alfalfa and oat.

**Items**	**OH**	**AH**	**SEM**	***P*-value**
pH_24h_	5.56	5.55	0.033	0.907
Dripping loss rate (%)	4.73	3.31	0.296	< 0.001
Cooking loss rate (%)	34.34	33.77	0.329	0.492
Moisture (%)	76.17	75.57	0.250	0.324
Protein (%)	21.00	20.50	0.142	0.111
Cholesterol (mg/100 g)	35.60	47.10	2.406	0.001
Intramuscular fat (%)	1.83	3.60	0.363	< 0.001
Ash (%)	0.85	0.76	0.020	0.019

Means within the same rows with different letters are significantly different (*p* < 0.05). SEM, standard error of means; OH, high oat percentages group; AH, high alfalfa percentage group.

FA components of the *longissimus lumborum* muscle of lambs allocated to each treatment group are shown in Table 3. Notably, the muscle fatty acid compositions of lambs were significantly (*p* < 0.05) influenced by the diet treatments. Differences in C14:0, C15:0, C16:0, C15:1, C16:0, C17:1, C18:0, C18:1n9t, C18:2n6t, C18:2n6c, C20:0, C21:0, and C22:0 content were not detected among the two diet treatments. Compared with the OH group, the AH group greatly (*p* < 0.05) increased the contents of C16:1, C17:0, and C20:3n6. The OH group expressed significantly higher (*p* < 0.05) C18:1n9c, C20:1, C18:3n3, and C22:6n3 content relative to the AH group. Finally, the MUFA contents were greatly (*p* < 0.05) reduced in the OH group, whereas the SFA and PUFA contents were not affected by diet treatment.

**Table 3:** Muscle fatty acid composition of longissimus lumborum muscle (mg/100 g of fatty acid methyl esters).

**Items**	**OH**	**AH**	**SEM**	***P*-value**
C14:0	4.55	5.99	0.904	0.529
C15:0	0.49	1.44	0.263	0.091
C15:1	0.25	0.13	0.079	0.557
C16:0	51.06	86.18	8.982	0.057
C16:1	3.77	5.98	0.554	0.049
C17:0	1.85	4.69	0.844	0.033
C17:1	1.95	1.94	0.010	0.547
C18:0	67.83	54.74	10.580	0.629
C18:1n9t	5.44	5.06	0.265	0.581
C18:1n9c	128.87	122.66	0.332	0.004
C18:2n6t	0.63	0.70	0.034	0.368
C18:2n6c	19.73	19.66	1.188	0.889
C20:0	0.32	0.57	0.092	0.253
C20:1	0.37	0	0.075	< 0.001
C18:3n3	1.66	1.22	0.101	0.022
C21:0	0.75	1.22	0.145	0.151
C22:0	0.54	0.54	0.037	0.984
C20:3n6	0	0.55	0.113	< 0.001
C22:6n3	0.47	0.38	0.018	< 0.001
SFA	128.83	153.92	10.339	0.318
PUFA	22.38	22.61	0.283	0.756
MUFA	140.53	135.89	1.021	0.008

Means within the same rows with different letters are significantly different (*p* < 0.05). SEM, standard error of means; OH, high oat percentages group; AH, high alfalfa percentage group; *c*, cis-fatty acid; *t*, trans-fatty acid.

### Rumen microbiota

**Figure 2:**
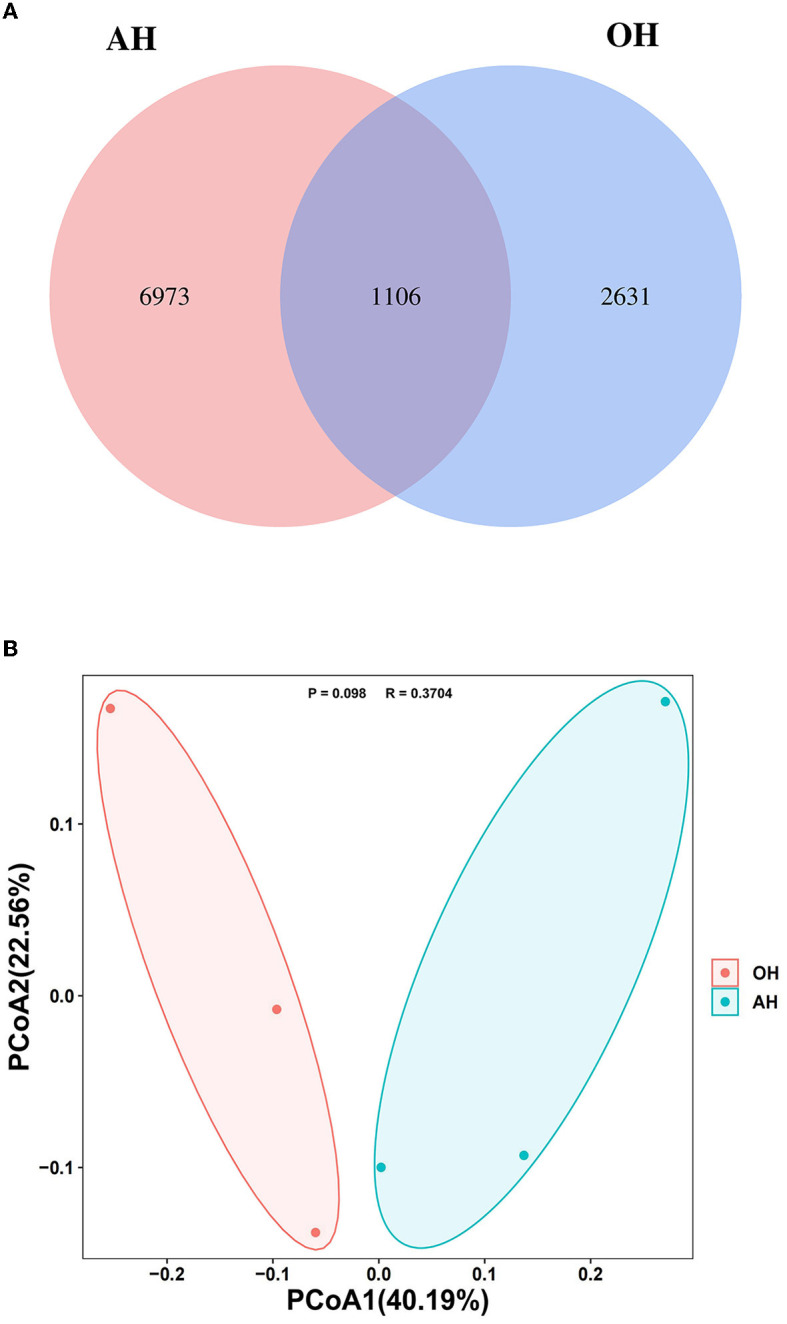
Microbial community among different treatments (*n* = 3). **(A)** Venn diagram representing the common and unique amplicon sequence variants (ASVs) found at each treatment. **(B)** Principal coordinates analysis (PCoA) of samples conducted based on weighted UniFrac distance. OH, high oat percentage group; AH, high alfalfa percentage group.

The Venn diagram of the rumen samples from the two FTMR diet groups revealed that a total of 11,816 ASVs were identified, and the groups shared 1,106 ASVs, whereas the OH and AH groups had 6,973 and 2,631 exclusive ASVs, respectively (Figure 2A).

The percentage of Good's coverage index for all samples in the two diet treatments was >99%. In the current study, the AH treatment increased the ASVs index and reduced the Shannon and Simpson indexes, but the differences were not significant (*p* > 0.05) (Table 4).

**Table 4:** Diversity indices of ruminal microbiota of lambs.

**Items**	**OH**	**AH**	**SEM**	***P*-value**
Observed_asvs (ASVs)	1,423	2,859	368.079	0.081
Shannon	9.58	8.59	0.245	0.141
Simpson	1.00	0.99	0.001	0.184
Chao1	3,141.20	1,525.10	415.820	0.086
Goods_coverage	0.99	0.99	0.003	0.184

Means within the same rows with different letters are significantly different (*p* < 0.05). SEM, standard error of means; OH, high oat percentage group; AH, high alfalfa percentage group.

PCoA plots with the weighted UniFrac distance metric revealed that the compositions of the bacterial community in the OH and the AH groups were basically distinguishable from each other (*R* = 0.3704, *P* = 0.098) (Figure 2B).

**Figure 3:**
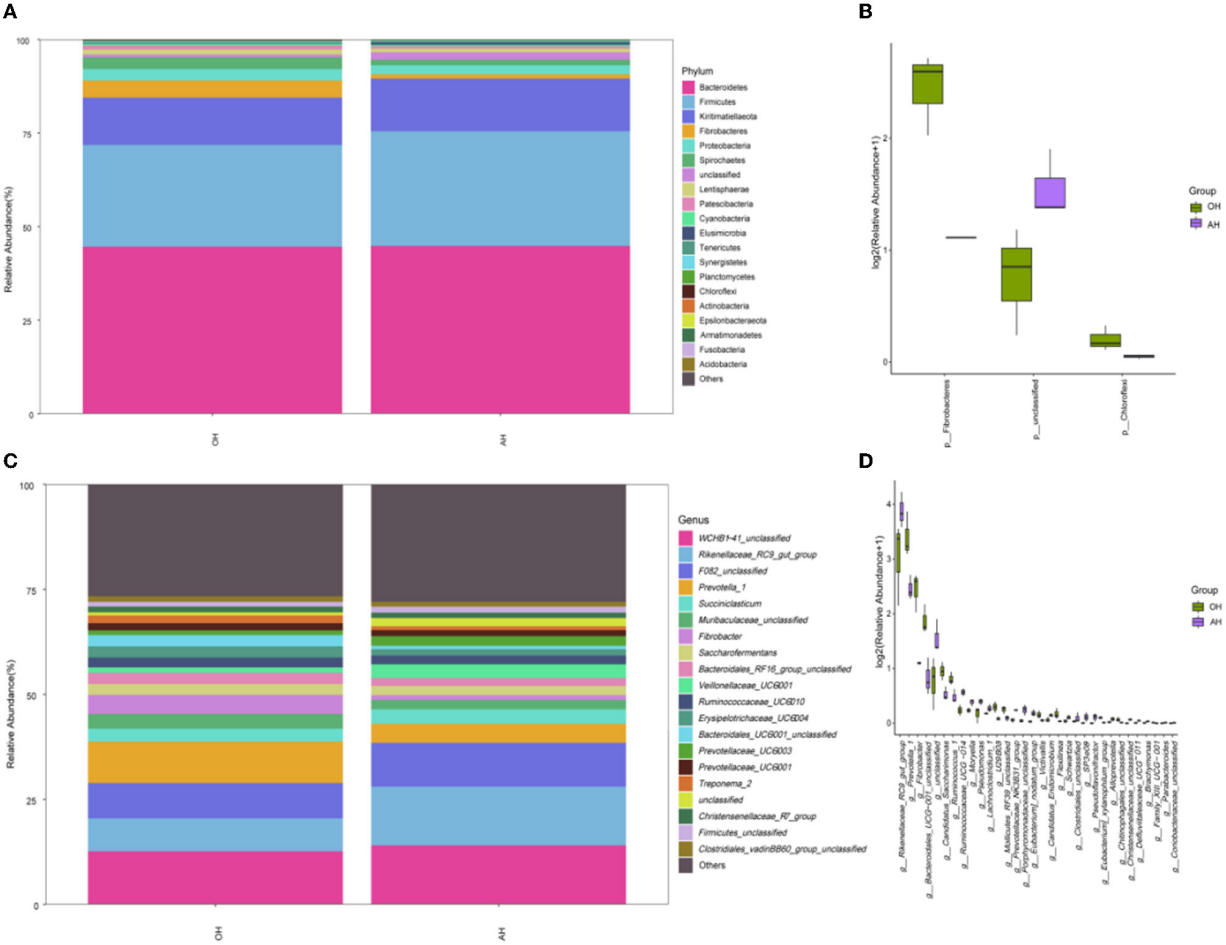
Relative abundance (%) of bacterial phyla (1% at least in one group) of ruminal microbiome of lambs fed FTMR with different alfalfa and oat ratios (*n* = 3). **(A)** Phylum level. **(B)** Extended error bar plot showing the bacteria at the phylum level that had significant differences among the AH and OH groups. **(C)** Genus level. **(D)** Extended error bar plot showing the bacteria at the genus level that had significant differences among the AH and OH groups. OH, high oat percentage group; AH, high alfalfa percentage group.

A list of rumen microbiota taxonomic distributions and relative abundances of the ruminal microbiota with a mean relative abundance of >1% and the top 20 at both phylum and genus levels are presented in Figure 3. Across all groups, 24 phyla and 413 genera were identified. At the phylum level, 15 bacteria phyla were detected (relative abundance > 1% at least in one group). The relative abundances in OH and AH groups revealed that Bacteroidetes (44.63 and 44.83%) was the most abundant bacterial phylum, followed by Firmicutes (27.18 and 30.66%), Kiritimatiellaeota (12.66% and 14.06%), Fibrobacteres (4.57 and 1.16%), Proteobacteria (3.08 and 2.40%), Spirochetes (3.12 and 1.48%), Lentisphaerae (1.36 and 1.01%) and Patescibacteria (1.04 and 0.62%), Cyanobacteria (0.48 and 0.39%), Elusimicrobia (0.11 and 0.63%), Tenericutes (0.34 and 0.25%), Synergistetes (0.27 and 0.24%), Planctomycetes (0.11 and 0.11%), and Chloroflexi (0.15 and 0.04%), respectively (Figure 3A). Abundances of Fibrobacteres and Chloroflexi were significantly (*p* < 0.05) different between the OH and AH groups (Figure 3B). At the genus level, 94 bacteria genera were thought to be the identifiable genera (relative abundance > 1% at least in one group). *WCHB1-41_unclassified* accounted for 12.66 and 14.06%, *Rikenellaceae_RC9_gut_group* accounted for 7.84 and 13.97%, *F082_unclassified* accounted for 8.40 and 10.43%, *Prevotella_1* accounted for 9.86 and 4.55%, and *Succiniclasticum* accounted for 3.09 and 3.44%, in the OH group and the AH group, respectively, and these were the dominant genera (Figure 3C). The OH and AH treatments resulted in significant (*p* < 0.05) differences in *WCHB1-41 unclassified, Rikenellaceae_RC9_gut_group* and F082_unclassified (Figure 3D).

**Figure 4:**
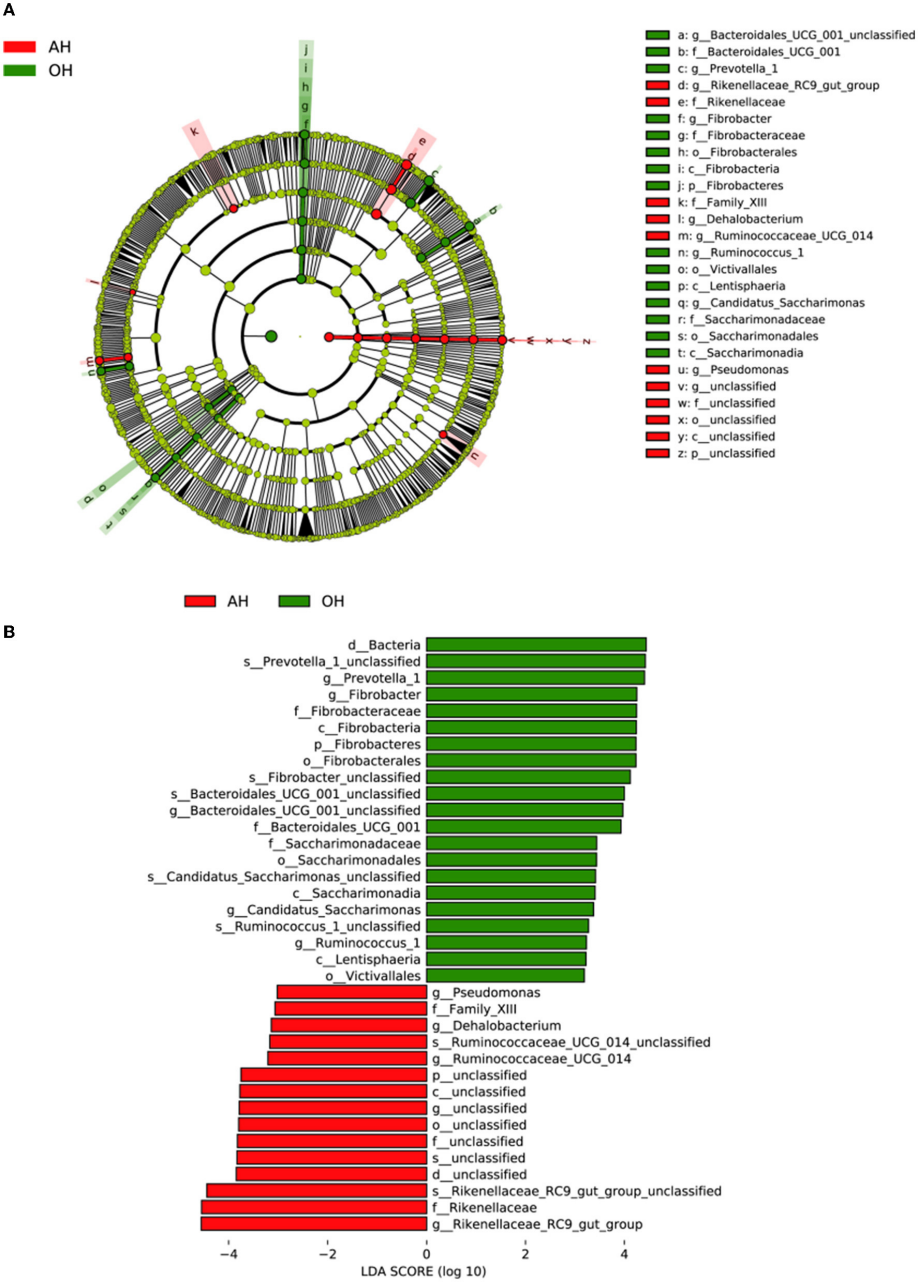
Linear discrimination analysis (LDA) coupled with effect size (LEfSe) analysis of the rumen microbial community of lamb in the AH and OH groups (*n* = 3). **(A)** Cladogram showing microbial species with significant differences among the two treatments. Red and green represent different groups. Species classification at the phylum, class, order, family, and genus level is displayed from inner to outer layers. The red and green nodes represent microbial species in the phylogenetic tree that play important roles in the AH and OH groups, respectively. Yellow nodes represent no significant difference between species. **(B)** Significantly different species with an LDA score greater than the estimated value (default score = 3). The length of the histogram represents the LDA score of different species in the two groups. OH, high oat percentage group; AH, high alfalfa percentage group.

Figure 4A shows a cladogram representative of the predominant bacteria and their structure, indicating that the most differentially abundant taxa enriched in the two groups. The results indicated that 15 clades were enriched in the OH group, and 11 clades were enriched in the AH group. The abundance differences among OH and AH were measured, and the results are shown in Figure 4B. The bacterial genus of *Prevotella_1, Fibrobacter, Bacteroidales_UCG_001_unclassified, Candidatus_Saccharimonas*, and *Ruminococcus_1* was the most enriched in the OH group, and the *Pseudomonas, Dehalobacterium, Ruminococcaceae_UCG_014*, and *Rikenellaceae_RC9_gut_group* were most enriched in the AH.

### Correlation analysis

**Figure 5:**
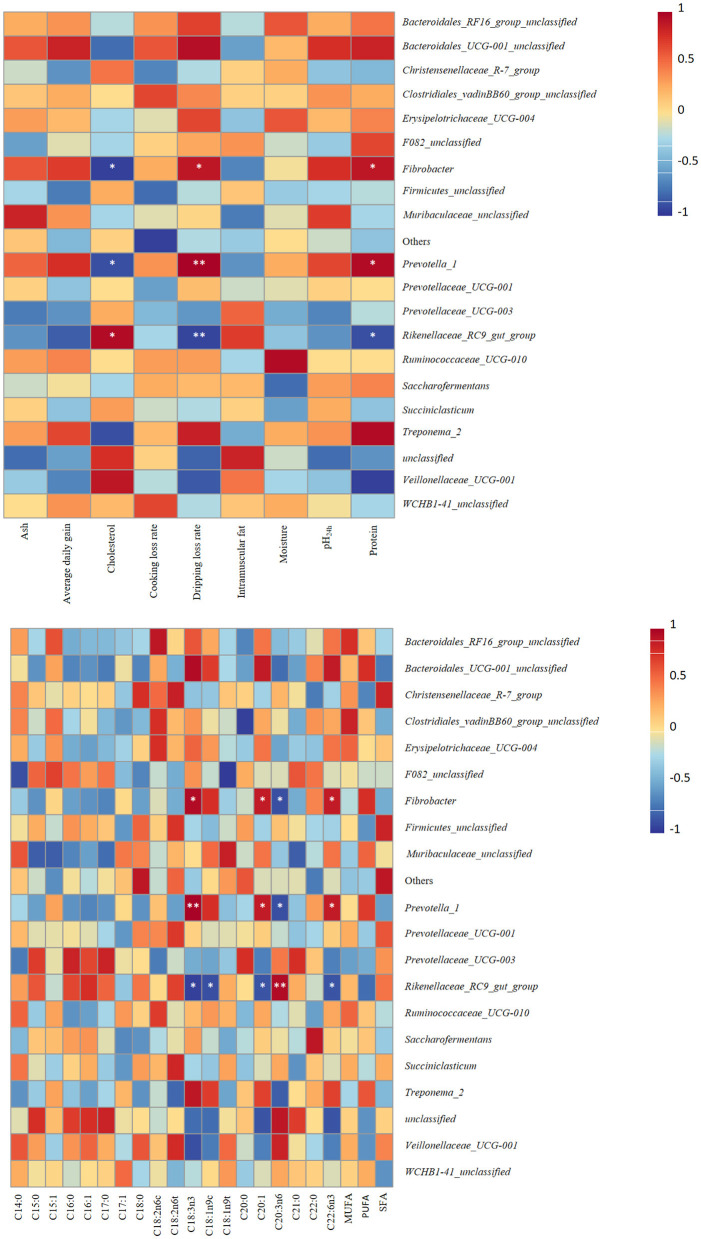
Heatmaps of Pearson's correlations between dominant genera and meat quality, and fatty acid profile. Red represents a positive correlation, while blue represents a negative correlation. Levels of significance are shown as follows: *P < 0.05; **P < 0.01.

Correlation analysis provided new insights into the confirmation of several bacterial genera potentially implicated in host development and meat quality improvement (Figure 5). Notably, the results revealed that the genera *Fibrobacter* and *Prevotella_1* were strongly correlated with dripping loss rate (*r* = 0.8986, *p* = 0.0149; *r* = 0.9856, *p* = 0.0003, respectively) and protein (*r* = 0.8857, *p* = 0.0333; *r* = 0.9429, *p* = 0.0167, respectively) but negatively correlated with cholesterol (*r* = −0.9429, *p* = 0.0167; *r* = −0.8857, *p* = 0.0333, respectively). The relative abundance of the *Rikenellaceae_RC9_gut_group* was positively correlated with cholesterol (*r* = 0.9429, *p* = 0.0167) but inversely correlated with dripping loss rate (*r* = −0.9276, *p* = 0.0167). The study findings support the idea that the genera *Fibrobacter* and *Prevotella_1* were positively related to C18:3n3 (*r* = 0.9429, *p* = 0.0167; *r* = 1.0000, *p* = 0.0028, respectively), C20:1 (*r* = 0.8783, *p* = 0.0213; *r* = 0.8783, *p* = 0.0213, respectively), and C22:6n3 (*r* = 0.8783, *p* = 0.0213; *r* = 0.8783, *p* = 0.0213, respectively) content but inversely correlated with C20:3n6 (*r* = −0.8804, *p* = 0.0206; *r* = −0.8804, *p* = 0.0206, respectively) content. The genera *Rikenellaceae_RC9_gut_group* was positively related to C20:3n6 (*r* = 0.9411, *p* = 0.0051) content but inversely correlated with C18:3n3 (*r* = −0.9429, *p* = 0.0167), C18:1n9c (*r* = −0.8804, *p* = 0.0206), C20:1 (*r* = −0.8783, *p* = 0.0213), and C22:6n3 (*r* = −0.8783, *p* = 0.0213) content. No significant correlation was found between the fat performance indexes and other changed genera whose abundance differed significantly between groups (*p* > 0.05).

### Predicted functional profiles

**Figure 6:**
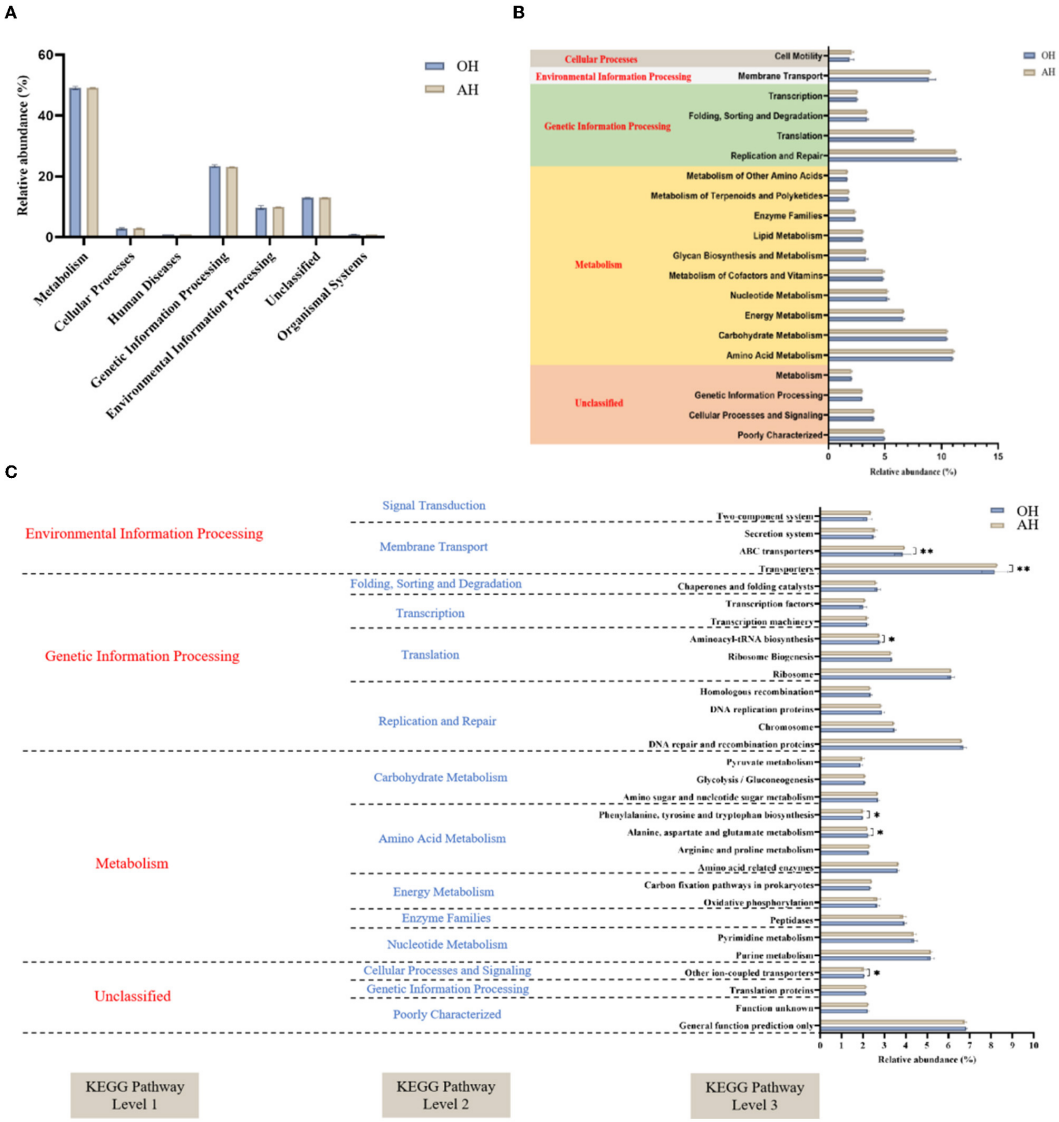
Dynamics of rumen bacterial predicted functional profiles fed with different diets analyzed by PICRUSt2 (*n* = 3). **(A)** Level 1 metabolic pathways. **(B)** Level 2 Kyoto Encyclopedia of Genes and Genomes (KEGG) ortholog functional predictions of the relative abundances of the top 20 metabolic functions. **(C)** Level 3 KEGG ortholog functional predictions of the relative abundances of the top 30 metabolic functions. OH, high oat percentage group; AH, high alfalfa percentage group. ^*^P < 0.05; ^**^P < 0.01.

To enable a better understanding of the functions of rumen microbiota, metabolic pathways of the microbiota involved were predicted according to the PICRUSt analysis using the KEGG pathway database (Figure 6). The primary predicted functional genes at level 1 in all groups were predominantly categorized into metabolism (48.60–49.66%), environmental information processing (23.04–23.87%), and genetic information processing (9.05–10.36%), respectively (Figure 6A). Among the enriched pathways, the relative abundance of replication and repair (11.35%), amino acid metabolism (11.01%), and carbohydrate metabolism (10.49%) accounted for more than 10% among the two groups. At the three levels, differences among the two groups in the microbial gene-predicted functions of bacteria are shown in Figure 6C. Notably, the genus associated with phenylalanine, tyrosine, and tryptophan biosynthesis, ABC transporters, and transporters were mainly accumulated in the AH group, while the genus correlated with alanine, aspartate, and glutamate metabolism and aminoacyl-tRNA biosynthesis were mainly expressed in the OH group.

## Discussion

Roughage serves as the major source of nutrients for ruminants and is potentially crucial for ruminant nutrient metabolism. An appropriate composition of roughage not only improves rumen microbiota but also promotes the rapid growth and development of ruminants (Cui et al., 2022). In the current research, the effect of FTMR diets with different forage proportions on fatty acid profile and rumen microbiota in lambs was investigated. The results provide a reference for promoting the formation of meat quality in lambs by regulating ruminal microbiota with roughage composition.

The results of this investigation indicated that forage proportion in FTMR had no adverse effects on the DMI of lambs, which is in agreement with Dung et al. (2020), who found the forage sources in FTMRs could directly reduce fiber characteristic effects on DM intake. Additionally, animals can adjust their feed intake behavior based on the energy level in the diet within a certain range (Wang et al., 2020). Thus, no significant difference was detected in DMI among the two groups due to their similar energy intake (Du et al., 2022). Furthermore, no remarkable difference was detected in ADG among AH and OH groups, which might be due to the similar DMI and energy intakes among the two groups (Mushi et al., 2009; Galvani et al., 2014).

The pH_24_ was kept within the normal range from 5.5 to 5.8 as suggested by Malva et al. (2016), which indicates that the lambs in this experiment did not have pre-slaughter animal stress and had a sufficient content of muscle glycogen. Intramuscular fat (IMF) is an important constituent of meat and affects its edibility (Brand et al., 2018). It has been demonstrated that the forage proportion in the diet affects the IMF content (Kotupan and Sommart, 2021). In the present investigation, intramuscular fat in lambs supplemented with AH was greater than that of the OH group. This result may be associated with the greater lactic acid bacteria amount in the diet of the AH group, which can produce the lactate to limit chylomicron secretion from enterocytes and lead to lipid synthesis and storage, a mechanism involving the conversion of lactate to malonyl-CoA, which subsequently inhibits β-oxidation and ultimately leads to IMF deposition after a series of reactions (Araujo et al., 2020). In addition, the increased lactic acid content in the AH group could be secondarily fermented in the rumen by lactate-utilizing bacteria to produce propionic acid (Li et al., 2021), resulting in increased fat deposition (Zhou et al., 2014). The significant difference between the two groups observed in cooking loss rates, dripping loss rates, and cholesterol could be explained by the difference in IMF content, and it has been shown that fat content affects meat palatability, including flavor and tenderness (Costa et al., 2012).

The component of fatty acids (FAs) in meat is crucial for the human health effects of meat consumption (Zhu et al., 2022). Meat from lamb is characterized by an abundance of saturated fatty acids (SFAs), monounsaturated and trans-fatty acids, and a low amount of polyunsaturated fatty acids (PUFA) (Behan et al., 2021). A previous study reported that unsaturated fatty acids can have positive effects on human health and the prevention of cardiovascular disease, and the excessive intake of saturated fatty acids can cause diseases such as arteriosclerosis (Shahidi and Ambigaipalan, 2018). The FA component of meat can be easily shaped by animal diets, as indicated by goats fed *Mitragyna speciosa* Korth leaves having more monounsaturated fatty acid than those fed Pangola grass hay (Chanjula et al., 2022). The intramuscular fat content of lambs fed OH had a higher C18:3n3 concentration than the AH group (*p* < 0.05). These differences may be interpreted as the difference in the FA compositions of AH and OH treatments because there is evidence that the content of C18:3n3 in oat hay is higher than that in alfalfa hay (Abidi et al., 2009; Whitney and Smith, 2015; Zhu et al., 2022). C18:3n3 is an important precursor of n-3 PUFA, especially the C22:6n3, which can promote the development of the nervous system and brain as an important raw material for the formation of biofilms (Youdim et al., 2000; Xu et al., 2019). In the current experiment, the results suggested that oat supplementation in FTMR diets improved the C22:6n3 content of lambs in terms of fatty acid nutrition, which was generally similar to the finding of Sun et al. (2021), who showed that C18:3n3 in muscle is a precursor substance for the synthesis of C22:6n3. C18:0 was the predominant SFA of lambs in the present study. The concentration of C18:0 was decreased in the AH diet, which might be because flavonoids in alfalfa played a certain role, as described by Su et al. (2022), who reported that the flavonoids of dietary alfalfa powder could decrease C18:0 content by regulating the activity of fatty acid metabolism-related enzymes. C18:1n9c was the main MUFA, as previously reported for lamb meat (Hajji et al., 2016). Significantly increased C18:1n9c content with the supplement of oat was observed in this study, which could be explained by the higher C18:0 content of the AH group that could form the C18:1n9c by the enzyme stearoyl Co-A desaturase (Wood et al., 2008). In addition, a higher concentration of MUFA was also found in the AH group, which may be due to the conversion of SFAs into MUFAs, especially the conversation of C18:0 into C18:1n9c (Sprecher et al., 1995).

Diet is a major factor in the formation of bacterial communities in the rumen (Huang et al., 2021). In this study, we evaluated the forage proportion in FTMR using information about ruminal bacteria based on high-throughput sequencing of the 16S rRNA. Differences in alpha diversity of ruminal bacteria were not detected among the diet treatments. This is consistent with some studies on ruminants (McGovern et al., 2018; McLoughlin et al., 2020). Similarly, Yang et al. (2018) also found that the diversity of ruminal bacteria was not affected by the forage type in Hu lambs. The current study showed that most of the two core phyla were Bacteroidetes and Firmicutes, which is consistent with previous studies (Derakhshani et al., 2017), in which Bacteroidetes and Firmicutes were found predominantly in rumen fluid. A previous study indicated that these two phyla were closely correlated with the decomposition of cellulose, hemicellulose, and polysaccharides (Liang et al., 2021). However, the phylum composition of Fibrobacteres was higher in the OH group relative to the AH group. Fibrobacteres play a primal function in the decomposition of fiber and cellulose to provide nutrients for ruminants (Chen et al., 2021). The current results may be interpreted as the higher fiber content of the OH group as described by An et al. (2020), who confirmed that the number of Fibrobacteres in the rumen tended to increase with an increase in coarse feed.

At the genus level, *WCHB1-41_unclassified, Rikenellaceae_RC9_gut_group, F082_unclassified*, and *Prevotella_1* were the dominant bacteria in the two diet groups. No differences were observed in *WCHB1-41_unclassified* and *F082_unclassified* bacteria among the two groups, but the OH group had a significant influence on the content of *Rikenellaceae_RC9_gut_group, Prevotella_1*, and *Fibrobacter* in rumen fluid. Although we do not know with certainty the function of *Rikenellaceae_RC9_gut_group*, a previous study has suggested that *Rikenellaceae_RC9_gut_group* is closely correlated with acetate and propionate (Clarke et al., 2013; Holman and Gzyl, 2019). Taken together, an increase in *Rikenellaceae_RC9_gut_group* in the AH group is suggestive of its major role in utilizing the carbohydrate and nitrogen in ruminants. This explains, partially at least, the improvement in IMF (Table 2). *Prevotella_1* is associated with carbohydrate metabolism and propionate production, which play a significant role in the synthesis and decomposition of plant non-cellulosic polysaccharides, starch, and protein (Liu et al., 2019; Zhang X. et al., 2021). The increase in the abundance of this genus in the AH group suggested that the higher percentage of oat in FTMR could promote the degradation both in fibrous and non-fibrous carbohydrates, and these differences may be due to responses to dietary nutrition changes, such as increased dietary starch (Liu et al., 2019), as has also been shown in previous research (Cui et al., 2022). *Fibrobacter* are cellulolytic bacteria, which specialize in cellulose and hemicellulose fermentation (Zhou et al., 2019; Wei et al., 2021). In this study, the AH group had a higher abundance of *Fibrobacter*, which may indicate its higher fiber content (Supplementary Table 1). This suggestion agrees with the finding of An et al. (2020), who reported that supplementing with oat hay in the diet promotes the decomposition of carbohydrates and dietary cellulose in the rumen, and then increased the content of *Fibrobacter*.

Correlation analysis revealed relationships between meat quality, fatty acid profile, and rumen microbial abundances. In this study, the AH treatment resulted in a higher abundance of *Rikenellaceae_RC9_gut_group* and higher cholesterol content, which is mainly due to the *Rikenellaceae_RC9_gut_group* belonging to the butyrate-producing bacteria, which could increase AMPK activity and further regulate lipid deposition traits by regulating the production of VFAs (Zhang Y. K. et al., 2021; Cheng et al., 2022). Furthermore, the increase in *Rikenellaceae_RC9_gut_group* abundance may affect C18:1n9c content (Figure 6), which can regulate cholesterol (Li et al., 2022). Ruminal microorganisms establish the key link between dietary nutrition and the fatty acids in ruminant products (Abuelfatah et al., 2016). Precursors for *de novo* fatty acid synthesis are mainly produced by rumen microbial fermentation (Shingfield et al., 2013). Therefore, ruminal rumen microbial metabolism is crucial in determining the ideal muscle fatty acid composition of ruminant products (Wang et al., 2022). A previous study has reported that reasonable muscle fatty acid compositions could be facilitated via the regulation of the bacterial community (Bi et al., 2018). The results of this study revealed that most parameters related to muscle FA deposition were correlated with the genera *Fibrobacter, Prevotella_1*, and *Rikenellaceae_RC9_gut_group*. C18:3n3 and C22:6n3 are the most bioactive components of ω-3 polyunsaturated fatty acids (PUFAs) and play an important role in reducing hepatic triglyceride content (Pirillo and Catapano, 2013). In this experiment, *Fibrobacter* and *Prevotella_1* were correlated positively with the C18:3n3 and C22:6n3 content. This result is mainly due to *Prevotella_1* and *Fibrobacter* being relevant for fiber digestion, which plays a crucial role in energy harvesting in the rumen ecosystem and provides precursors for UFA synthesis (Abuelfatah et al., 2016; Wang et al., 2019). Similar findings have been reported in fattening yaks (Hu et al., 2020). *Rikenellaceae_RC9_gut_group* was negatively correlated with the concentrations of C18:3n3 and C22:6n3. This result may be explained by the *Rikenellaceae RC9 gut group* having important metabolic functions in lipid metabolism (Ahmad et al., 2020). Research has shown that the *Rikenellaceae_RC9_gut_group* is negatively correlated with propionic acid (Cheng et al., 2022), which is produced by the biohydrogenation of PUFA (Beam et al., 2000). Furthermore, a previous study has reported that n-3 PUFA deficiency increased *Rikenellaceae* (Leyrolle et al., 2021).

Microorganisms are important in modulating the host's adaptive immunity and regulating the degradation, distribution, and absorption of nutrients (Fan et al., 2020).

In the current research, the predicted function from the level 1 gene was generally consistent among the two diet groups. However, the observations indicated that the diets directly impacted the levels 2 and 3 estimated gene functional profiles of lamb rumen microbiomes. Compared to the AH group, the metabolism of amino acids, including phenylalanine, tyrosine, and tryptophan biosynthesis, was decreased in the OH group. These results may be interpreted as due to the significant enrichment of *Prevotella_1* and *Fibrobacter* regulating the rumen protein metabolism and cellulose and hemicellulose metabolism and favoring the preferential use of carbohydrates, which would then weaken amino acids fermentation (Zhang et al., 2022). Hence, the pathways of “phenylalanine, tyrosine, and tryptophan biosynthesis” identified in this experiment may be attributed to the higher fiber content in the FTMR of lambs fed OH diets. However, our study findings do not represent the actual function of rumen bacteria. Further detailed analyses are needed to investigate the potential mechanisms and reveal the gene functions of microbiota under diets with different forage sources in FTMR fed to lambs.

## Conclusion

This preliminary investigation explored the effect of forage proportions in FTMR on fatting performance and the rumen bacterial community of lambs. The fatting performance analysis showed that partial replacement of alfalfa with oat in FTMR promotes a beneficial lipid pattern in the *Longissimus lumborum* muscles for lambs. In addition, the correlation analysis revealed that there was a strong correlation between specific rumen bacteria and fatty acids in the *Longissimus lumborum* muscles. Of course, the lack of rumen fermentation parameters and quantification of probiotics as well as the small sample size are potential limitations of this study. Regardless, the results of the current research suggested that oat-based FTMR was more conducive to the production of beneficial fatty acids in the *Longissimus lumborum* muscles for lambs. These results can also provide some references for the application of FTMR in animal production, and information on interactions of the fatting performance of lamb and microbiota of the rumen, which may help make decisions regarding feeding.

## Data availability statement

The datasets presented in this study can be found in online repositories. The names of the repository/repositories and accession number(s) can be found below: https://www.ncbi.nlm.nih.gov/, PRJNA888233.

## Ethics statement

The study protocol selected for this experimental was based on the Institutional Guidelines for Animal Experiments and the Regulations for the Administration of Affairs Concerning Experimental Animals of the College of Grassland, Resources and Environment, Inner Mongolia Agricultural University, Hohhot, China. All the experimental protocols carried out in this study were approved by the Animal Care Committee of Inner Mongolia Agricultural University. Written informed consent was obtained from the owners for the participation of their animals in this study.

## Author contributions

ML: conceptualization, methodology, data curation, writing—original draft preparation, and writing—reviewing and editing. ZW: investigation and resources. YW: methodology. LS: software. JL: validation and formal analysis. GG: writing—reviewing and editing. YJ: project administration and funding acquisition. SD: writing—original draft preparation and writing—reviewing and editing. All authors have read and agreed to the published version of the manuscript.
