# Supplementary material for: Effects of different forage proportions in fermented total mixed ration on muscle fatty acid profile and rumen microbiota in lambs
Source: Front Microbiol. 2023 Jul 13;14:1197059. doi: 10.3389/fmicb.2023.1197059 (PMC10374311; doi:10.3389/fmicb.2023.1197059)
Supplement: Supplementary file 1 [file Table_1.DOCX]

***Supplementary Material***

**Effects of Different Forage Types in Fermented Total Mixed Ration on Muscle Fatty Acid Profile and Rumen Microbiota in Lambs**

**Mingjian Liu^1^, Zhijun Wang^1^, Lin Sun^2^, Yu Wang^1^, Junfeng Li^3^, Gentu Ge^1^, Yushan Jia^1,*^, Shuai Du^1*^**

*Correspondence: [jys_nm@sina.com;](mailto:jys_nm@sina.com;) [dushuai_nm@sina.com](mailto:dushuai_nm@sina.com)

1. **Supplementary Data**

**Supplementary** **TABLE 1 |** Ingredients and chemical composition of dietary

| Items | OH | AH |
| --- | --- | --- |
| Ingredient (g/kg DM) |  |  |
| Oat hay | 350 | 250 |
| Alfalfa hay | 250 | 350 |
| Natural forage | 30 | 30 |
| Corn stalk | 20 | 20 |
| Corn | 200 | 230 |
| Soybean meal | 110 | 80 |
| Wheat bran | 20 | 20 |
| Calcium hydrogen phosphate | 3 | 3 |
| Nacl | 2 | 2 |
| NaHCO_3_ | 5 | 5 |
| Premix | 10 | 10 |
| Chemical compositions |  |  |
| DM (g/kg FM) | 45.40±0.53 | 45.75±0.34 |
| CP (g/kg DM) | 13.05±0.20 | 13.23±0.19 |
| NDF (g/kg DM) | 51.79±0.01a | 48.25±0.01b |
| ADF (g/kg DM) | 32.20±0.01 | 30.80±0.01 |
| OM (g/kg DM) | 34.02±0.69 | 35.16±0.60 |
| ME (MJ/kg) | 10.88±0.01 | 10.59±0.01 |
| Fermentation profile |  |  |
| pH | 4.67±0.02 | 4.61±0.03 |
| Lactic acid (g/kg DM ) | 9.95±0.48 | 10.27±0.15 |
| Acetic acid (g/kg DM ) | 0.89±0.05 | 0.85±0.03 |
| Propionic acid (g/kg DM ) | 0.63±0.05b | 1.05±0.02a |
| Ammonia-N (g/kg DM ) | 2.75±0.22 | 3.30±0.14 |
| Microbial counts |  |  |
| Lactic acid bacteria (Log_10_ cfu/g FM) | 6.07±0.67 | 7.13±0.40 |
| Aerobic bacteria (Log_10_ cfu/g FM) | 6.48±0.24 | 5.40±0.50 |

DM, dry matter; FM, fresh matter; CP , crude protein; NDF, neutral detergent fiber; ADF, acid detergent fiber; OM, organic matter; ME, metabolizable energy. Composition of mineral premix. Per kg: Copper 1800 mg, iron 3400 mg, manganese 1500 mg, zinc 1700 mg, cobalt 20 mg, vitamin A 1620 000 IU, vitamin D332 400 IU, vitamin E 540 IU, folic acid 15 mg. OH, high oat percentages group; AH, high alfalfa percentages group.
